# Long-Term Postoperative Patient-Reported Quality of Life After Endoscopic Surgery for Sinonasal Inverted Papilloma: A Longitudinal Cohort Study

**DOI:** 10.3390/medicina62081567

**Published:** 2026-08-15

**Authors:** Marcelina Niemiec-Urbańczyk, Maria Humeniuk-Arasiewicz, Karolina Goroszkiewicz, Grażyna Stryjewska-Makuch, Joanna Glück

**Affiliations:** 1 Department of Laryngology and Laryngological Oncology, Upper-Silesian Medical Centre, Medical University of Silesia, 40-635 Katowice, Poland; 2 Department of Otorhinolaryngology and Laryngological Oncology in Zabrze, Medical University of Silesia, 41-800 Katowice, Poland; 3 Department of Internal Diseases, Allergology and Clinical Immunology in Katowice, Medical University of Silesia, 40-752 Katowice, Poland

**Keywords:** papilloma, inverted, quality of life, Sino-Nasal Outcome Test, patient-reported outcome measures, cohort studies

## Abstract

*Background and Objectives*: Sinonasal inverted papilloma (IP) is a benign yet locally aggressive tumour associated with a clinically relevant risk of recurrence and malignant transformation. While surgical outcomes are well documented, data on long-term patient-reported quality of life (QoL) remain limited and are often based on short-term or single time-point assessments using disease-specific instruments alone. *Materials and Methods*: We conducted a single-centre observational cohort study of adults with histologically confirmed IP treated endoscopically. Patients were followed in a university-affiliated otorhinolaryngology outpatient clinic and completed the Sino-Nasal Outcome Test-22 (SNOT-22) at each visit; from 2022 onwards, the generic WHOQOL-BREF questionnaire was additionally administered. Longitudinal SNOT-22 trajectories were analysed using generalised additive models and linear mixed-effects models. In a subgroup of patients, retrospectively recalled preoperative SNOT-22 and WHOQOL-BREF scores were used only for exploratory paired pre–post comparisons. *Results*: Sixty-nine patients were included. Mean follow-up was 63.1 ± 27.8 months, and recurrence occurred in 21 of 69 patients (30%). A total of 461 postoperative SNOT-22 assessments with complete follow-up time data were included in the longitudinal analysis, with a median of six assessments per patient. Mean postoperative SNOT-22 score was 25.4 ± 19.8, and longitudinal postoperative trajectories showed a largely stable course over time. In the paired exploratory subgroup (*n* = 34), retrospectively recalled preoperative SNOT-22 scores were higher than postoperative scores, although these exploratory findings should be interpreted cautiously because baseline PROMs were not collected prospectively. Exploratory paired comparisons of WHOQOL-BREF showed higher postoperative scores in the physical, psychological and environmental domains. *Conclusions*: Patients with sinonasal IP treated endoscopically can achieve favourable and largely stable long-term disease-specific and generic QoL. No single baseline clinical factor showed a clear association with postoperative SNOT-22 scores in the longitudinal models. Combining disease-specific and generic patient-reported outcome measures with longitudinal modelling may support more informed postoperative counselling in this rare, recurrent condition, while structured surveillance remains essential.

## 1. Introduction

Inverted papilloma (IP) is a benign epithelial tumour arising from the Schneiderian mucosa of the nasal cavity and paranasal sinuses, accounting for approximately 0.5–4% of tumours in this region, with an estimated incidence of 0.2–1.5 per 100,000 person-years [1,2]. Despite its benign histology, sinonasal inverted papilloma (IP) is a locally aggressive tumour that may cause bone remodelling, erosion and extension within the sinonasal tract [1,2]. Recurrence remains clinically relevant, although reported rates vary according to tumour extent, attachment site, previous surgery, surgical approach and follow-up duration [3,4,5]. Contemporary endoscopic series generally report lower recurrence rates than historical or revision cohorts, but late recurrence may still occur years after apparently complete resection [3,4,5]. IP also carries a recognised risk of malignant transformation to squamous cell carcinoma (SCC), most commonly reported at approximately 5–15%, with higher rates in selected series [6,7,8]. Therefore, long-term ENT surveillance, based mainly on endoscopy and imaging when indicated, remains essential; serum SCC antigen may provide additional value in selected patients [1,9].

Diagnosis may be further complicated by overlap with chronic rhinosinusitis with nasal polyps (CRSwNP). Radiological inflammatory changes and bilateral polyps do not exclude IP, as 17–23% of IP cases coexist with sinonasal polyps [2]. Consequently, IP may remain under-recognised or be misattributed to inflammatory disease, particularly when symptoms are intermittent rather than persistent.

Patient quality of life (QoL) is now regarded as a key outcome alongside traditional clinical endpoints [10]. The World Health Organization defines QoL as an individual’s perception of their position in life within their cultural and value context and in relation to their goals, expectations, standards and concerns [11]. Schipper introduced the concept of health-related quality of life (HRQoL), reflecting the impact of disease and its treatment as perceived by the patient across somatic, psychological, functional and social domains [12]. Patient-reported outcome measures (PROMs) capture these dimensions and may be generic or disease-specific [13]. WHOQOL-BREF is a widely used generic HRQoL instrument developed by the World Health Organization [11], validated in Polish by Jaracz et al. [14], and previously applied in rhinologic populations [15].

Most QoL data in sinonasal IP have relied on the disease-specific SNOT-22 questionnaire, a well-established patient-reported outcome measure [16,17,18]. Several single-centre and prospective studies have used SNOT-22 to assess postoperative outcomes in patients with sinonasal IP [19,20,21,22]. While these studies generally report favourable postoperative outcomes, the evidence remains limited by relatively short or mid-term follow-up in many cohorts and infrequent assessment of generic HRQoL [19,20,21,22]. A recent review by Chow et al. highlighted that QoL research in sinonasal tumours remains fragmented and that generic and disease-specific instruments are rarely combined [23]. The 2024 International Consensus Statement on Sinonasal Tumours likewise emphasises the lack of validated tumour-specific QoL tools and recommends combining generic and disease-specific PROMs whenever feasible [24]. Taken together, the available evidence suggests generally favourable postoperative quality of life; however, long-term longitudinal studies based on repeated patient-reported outcome assessments remain scarce, particularly beyond five years of follow-up.

The primary aim of this study was to characterise long-term trajectories of disease-specific QoL in patients with sinonasal IP treated endoscopically, using repeated SNOT-22 measurements over time. As a key secondary objective, we described long-term generic HRQoL using WHOQOL-BREF and contextualised postoperative domain scores against available Polish normative data. As prospectively collected preoperative PROMs were unavailable, retrospectively recalled preoperative SNOT-22 and WHOQOL-BREF scores were used only as exploratory comparators in a subset of patients.

## 2. Materials and Methods

### 2.1. Study Design and Population

This single-centre observational cohort study was conducted at the Department of Otorhinolaryngology of a university-affiliated hospital in Poland. The study combined retrospective and prospective components. Clinical, surgical, and follow-up data were collected retrospectively from medical records, whereas postoperative patient-reported outcome measures (PROMs) were collected prospectively during routine follow-up visits. Retrospectively recalled preoperative SNOT-22 and WHOQOL-BREF scores were obtained during follow-up using a then-test approach and were used exclusively for exploratory paired pre–post comparisons. This study was reported in accordance with the Strengthening the Reporting of Observational Studies in Epidemiology (STROBE) statement for cohort studies (File S1). Systematic follow-up of patients with IP started in March 2018 and was scheduled at 3–6-month intervals. Follow-up data for the present study were collected through 30 September 2025. All adults undergoing surgical treatment for IP at our centre from 1 January 2016 onwards were screened for eligibility based on histopathological confirmation and review of clinical records.

**Figure 1:**
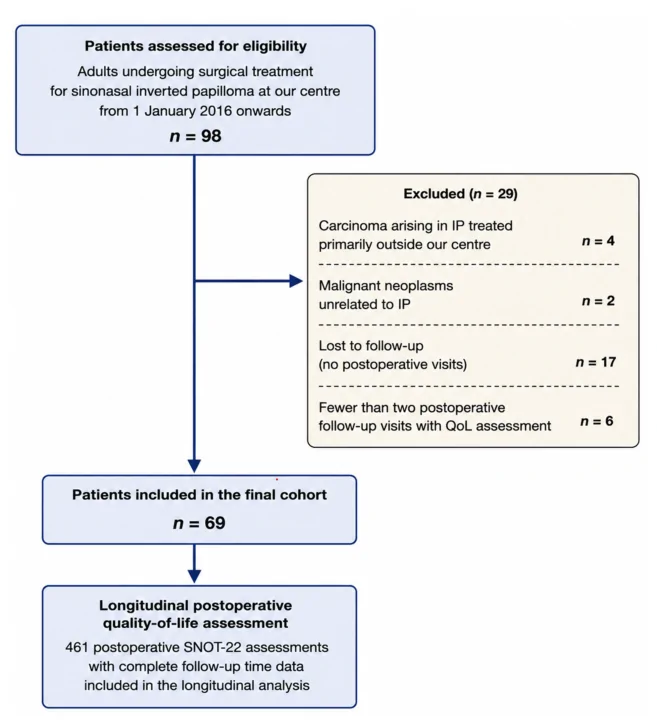
Flowchart of patient selection and inclusion in the longitudinal cohort.

A total of 98 patients were initially identified. Exclusion criteria were: (1) carcinoma arising in IP treated primarily outside our centre (*n* = 4), (2) malignant neoplasms unrelated to IP (*n* = 2), (3) loss to follow-up (no postoperative visits; *n* = 17), and (4) fewer than two postoperative follow-up visits with QoL assessment (*n* = 6). The final study cohort comprised 69 patients. The study size was determined by the number of eligible patients meeting the study criteria; no a priori sample size calculation was performed (Figure 1).

### 2.2. Follow-Up Protocol and Data Collection

Patients were routinely scheduled for postoperative follow-up visits every 3–6 months in the otorhinolaryngology outpatient clinic. At each visit, a structured medical history was obtained, nasal endoscopy was performed, and available imaging (CT/MRI) was reviewed to evaluate for residual or recurrent disease. In routine clinical practice, the actual timing of some visits varied because of patients’ individual scheduling needs and temporary healthcare disruptions associated with the COVID-19 pandemic.

From 2018 onwards, patients were routinely asked to complete the SNOT-22 questionnaire at each visit; from 2022 onwards, the WHOQOL-BREF questionnaire was additionally administered. Questionnaires were completed independently after brief instructions from a physician and checked for completeness in the patient’s presence.

For the present analyses, we included only visits with complete SNOT-22 data and patients with at least two postoperative visits. Follow-up time was calculated in months from the date of index surgery.

Preoperative SNOT-22 and WHOQOL-BREF scores were not collected prospectively at the time of surgery. In 34 of 69 patients, retrospectively recalled preoperative scores were obtained during follow-up visits using a retrospective pre-test/“then-test” approach, whereby patients were asked to rate their perceived status immediately before surgery. Given the risk of recall bias and response-shift bias, these retrospectively recalled preoperative PROMs were used only for exploratory paired pre–post comparisons and were not included in the main longitudinal models.

### 2.3. Quality-of-Life Instruments

SNOT-22 comprises 22 items assessing sinonasal and related symptoms, each scored from 0 (“no problem”) to 5 (“worst possible problem”), yielding a total score of 0–110; higher scores indicate worse disease-specific QoL [16,17,18]. A Polish-language version of the SNOT-22 was used throughout the study. The same version subsequently underwent formal linguistic adaptation and validation in Polish-speaking patients, with satisfactory reliability and validity reported by Morawska et al. in 2024 [25].

WHOQOL-BREF is a generic HRQoL instrument developed by the World Health Organization. It includes 26 items covering four domains (physical, psychological, social relationships and environment) and two global items on overall QoL (Q1) and general health (Q2) [11,14]. The Polish version has been validated by Jaracz et al. [14]. Domain scores were calculated according to WHO guidelines, with higher scores indicating better HRQoL. Permission to use the questionnaire was obtained from WHO (no. 380625). For contextualisation, postoperative WHOQOL-BREF domain scores in our cohort were compared descriptively with normative data for Polish working-age adults from an industrial region reported by Kowalska et al. [26].

The primary outcome was the long-term course of disease-specific QoL measured by repeated SNOT-22 assessments. Long-term generic HRQoL measured with WHOQOL-BREF was a key secondary outcome. Retrospectively recalled preoperative SNOT-22 and WHOQOL-BREF scores were considered exploratory secondary comparators and were not treated as equivalent to prospectively collected baseline PROMs.

### 2.4. Clinical Variables

Baseline variables included age, sex, smoking status (current or former vs. never), Krouse stage (T1–T4) [1], side (left/right), tumour location (maxillary sinus, ethmoid, nasal cavity, frontal sinus, septum, sphenoid sinus), history and number of previous sinonasal operations, and surgical approach (endoscopic, classical polypectomy, external/open, debulking). Time to recurrence was defined as the interval from index surgery to the date of endoscopic and/or radiological diagnosis of recurrent IP.

### 2.5. Statistical Analysis

Analyses were performed in R. Continuous variables are presented as mean ± SD or median (IQR), as appropriate; categorical variables are presented as counts and percentages. Between-group comparisons were performed using Welch’s *t*-test for continuous variables and Fisher’s exact test for categorical variables. Statistical significance was set at *p* < 0.05.

To describe longitudinal SNOT-22 trajectories, we fitted generalised additive models (GAMs) with smoothing splines for time (months since surgery) and 95% confidence intervals for the mean. Models were fitted for the overall cohort and stratified by selected clinical variables (e.g., sex, smoking, previous surgery, recurrence, side) and visualised as scatterplots with overlaid smoothed curves.

For inferential longitudinal analyses, we used linear mixed-effects models (LMMs) with a patient-level random intercept to account for within-patient correlation. Time was modelled as a continuous variable, and the coefficient for time was scaled to represent the mean 6-month change in SNOT-22 (β < 0 indicating improvement; β > 0 indicating worsening). We first fitted univariable LMMs including time and one clinical covariate at a time (sex, smoking, Krouse stage, previous surgery, number of previous operations, side, location). Estimates were obtained using restricted maximum likelihood (REML), with *p*-values based on Satterthwaite’s approximation and 95% Wald confidence intervals. Exploratory subgroup analyses and paired pre–post comparisons of SNOT-22 and WHOQOL-BREF in the paired subgroup were performed using the Wilcoxon signed-rank test. These paired comparisons were interpreted as exploratory only because the preoperative values were retrospectively recalled rather than prospectively collected. Linear mixed-effects models included a patient-level random intercept to account for within-patient correlation of repeated measurements. Alternative models including a random slope for time were evaluated but did not improve model fit (likelihood-ratio test: χ^2^(2) = 3.03, *p* = 0.22; ΔAIC = +0.57; ΔBIC = +8.85); therefore, the more parsimonious random-intercept model was retained. Time since surgery was modelled as a continuous variable, rescaled into 6-month units and mean-centred without standardisation. Model assumptions were assessed graphically by inspection of residual-versus-fitted plots, quantile–quantile plots of conditional residuals and random effects, and Cook’s distance. No substantial deviations from model assumptions were identified. Missing data were not imputed. The mixed-effects modelling framework accommodated the unequal number and timing of repeated measurements across patients. Linear mixed-effects models were fitted using all available postoperative observations under the assumption that data were missing at random (MAR); patients with missing values for a specific clinical covariate were excluded only from analyses involving that covariate.

### 2.6. Ethics

All patients provided written informed consent to participate in the follow-up programme and to complete QoL questionnaires. The study was conducted in accordance with the Declaration of Helsinki. The Local Bioethics Committee issued a formal statement that, given the retrospective observational nature of the analysis, separate ethical approval was not required (No. CN/CBN/0052/KB/181/22).

## 3. Results

### 3.1. Baseline Characteristics

Sixty-nine patients with sinonasal inverted papilloma were included (Table 1). The cohort was predominantly in Krouse stages T2–T3, and nearly half had undergone at least one previous sinonasal operation. Mean follow-up exceeded five years, and recurrence was observed in 21 of 69 patients (30%). Median follow-up was 67 months (IQR 35–88; range 16–115). Follow-up of at least 24, 36 and 60 months was available in 61/69 (88.4%), 50/69 (72.5%) and 42/69 (60.9%) patients, respectively. Baseline characteristics stratified by sex, smoking status, side, previous surgery and recurrence are provided in Table 1 and Supplementary Tables S1–S5.

### 3.2. Longitudinal Course of SNOT-22

**Figure 2:**
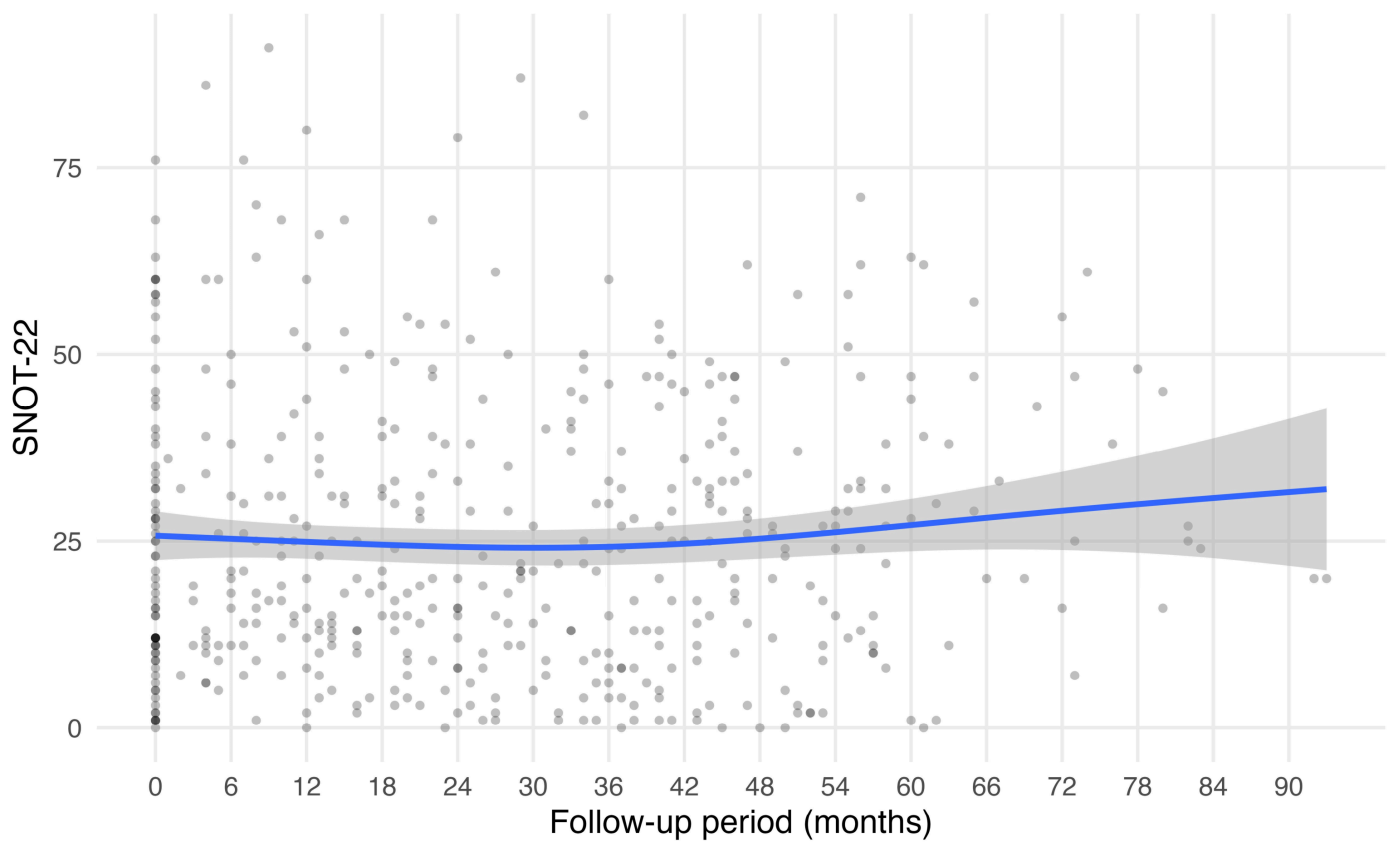
Longitudinal SNOT-22 scores after endoscopic surgery for sinonasal inverted papilloma. Each dot represents an outpatient visit. The solid line indicates the fitted GAM trajectory and the shaded area the 95% confidence interval. Time since surgery (months) is shown on the *x*-axis and SNOT-22 score on the *y*-axis.

Across all postoperative visits, SNOT-22 scores indicated a mild-to-moderate symptom burden (Table 1). A total of 461 postoperative SNOT-22 assessments with complete follow-up time data were included in the longitudinal analysis, with a median of six assessments per patient (IQR 4–10; range 2–11). GAM-based postoperative trajectories showed a largely stable course during follow-up, without evidence of progressive deterioration (Figure 2). Stratified trajectories by sex, smoking status, previous surgery, recurrence and side (Supplementary Figures S1–S5) were broadly similar, with overlapping confidence intervals. Patients with recurrence did not demonstrate a distinct pattern of longitudinal SNOT-22 deterioration compared with those without recurrence, although these subgroup analyses should be interpreted cautiously because of the limited number of recurrent cases.

In univariable LMMs, the time coefficient (scaled per 6 months) did not suggest progressive worsening of SNOT-22 in the overall cohort. None of the evaluated demographic or clinical variables—including sex, smoking status, Krouse stage, previous surgery, number of previous operations, side or location—showed a statistically significant association with postoperative SNOT-22 scores after accounting for follow-up time (Table 2). Overall, these analyses suggest that disease-specific QoL remains largely stable over time, with no single baseline clinical factor showing a clear association with postoperative SNOT-22 scores.

Linear mixed-effects models with random intercept at patient level; time treated as a continuous variable (months since surgery), with β coefficients scaled to represent the mean change in SNOT-22 per 6 months (β < 0 indicates improvement; β > 0 worsening). Models estimated using restricted maximum likelihood (REML); *p*-values based on Satterthwaite’s approximation; 95% Wald confidence intervals. For categorical variables, effects are presented relative to the reference category.

### 3.3. Quality of Life: SNOT-22 and WHOQOL-BREF

Prospectively collected preoperative PROMs were not available. Retrospectively recalled preoperative SNOT-22 and WHOQOL-BREF scores were available in the paired exploratory subgroup; therefore, all paired pre–post comparisons should be considered exploratory and should not be interpreted as equivalent to analyses based on prospectively collected baseline PROMs.

In the paired subgroup (*n* = 34), postoperative SNOT-22 scores were lower than retrospectively recalled preoperative scores (Table 3). In the same subgroup, postoperative WHOQOL-BREF scores were higher in the physical, psychological and environmental domains, while the social relationships domain remained unchanged; differences in the two global items were not statistically significant (Table 3, Table S6).

Exploratory subgroup analyses by sex, smoking status, previous surgery and recurrence (Supplementary Tables S7–S10) suggested broadly similar directions of change, although statistical significance was not uniform and should be interpreted cautiously due to limited sample size and multiple testing.

When contextualised descriptively against normative Polish WHOQOL-BREF data reported by Kowalska et al. [26], postoperative IP patients achieved HRQoL levels comparable to, and in some domains slightly higher than, those of working-age adults from an industrial region. This indirect comparison should be interpreted with caution.

## 4. Discussion

In this single-centre cohort, long-term disease-specific QoL after endoscopic surgery for sinonasal inverted papilloma was generally favourable and remained stable during extended follow-up. Longitudinal modelling did not indicate progressive symptom deterioration over time, and no evaluated demographic or tumour-related factor showed a clear association with postoperative SNOT-22 scores after accounting for follow-up time. In the subgroup with retrospectively recalled preoperative PROMs, exploratory paired comparisons suggested lower postoperative SNOT-22 scores and higher WHOQOL-BREF domain scores compared with retrospectively recalled baseline values. Because preoperative PROMs were not prospectively collected, these findings should be interpreted as supportive and hypothesis-generating, whereas the main finding of the study is the favourable and stable long-term postoperative QoL trajectory.

Our findings align with prior reports indicating that QoL after endoscopic treatment of IP is generally good and may approach reference population levels. Abiri et al. and van Samkar and Georgalas reported sustained postoperative improvement in disease-specific QoL, whereas den Heijer et al. highlighted the role of baseline symptom burden and systemic comorbidity in shaping HRQoL trajectories [19,20,21]. By combining repeated SNOT-22 assessments with longitudinal modelling and complementing these data with WHOQOL-BREF, our study adds longer-term follow-up and a broader HRQoL perspective contextualised to Polish normative data. However, comparisons with published normative WHOQOL-BREF data should be regarded as descriptive only, because the reference populations are not directly comparable with our cohort with respect to age, health status, and other demographic and clinical characteristics.

Most previous QoL research in IP has relied on SNOT-22 alone. While SNOT-22 is well validated and widely used, it emphasises sinonasal symptoms and captures broader HRQoL domains only indirectly [16,17,18]. Recent reviews and consensus documents underscore the lack of tumour-specific QoL tools and support combining disease-specific and generic PROMs where feasible [23,24]. In this context, WHOQOL-BREF provided complementary information, suggesting that favourable postoperative functioning was observed across physical, psychological and environmental domains, although these findings should be interpreted in light of the exploratory design of the pre–post analyses. However, because preoperative WHOQOL-BREF scores were retrospectively recalled, domain-level pre–post changes should be interpreted cautiously and require confirmation in prospective cohorts with baseline PROM collection.

Although recurrence was not associated with clearly higher postoperative SNOT-22 scores in this cohort, symptom burden and tumour recurrence do not necessarily correlate directly. Recurrent inverted papilloma may not be accompanied by clinically apparent changes in sinonasal symptoms at the time of detection and may instead be identified during scheduled endoscopic surveillance. Therefore, symptom-based PROMs should complement, but not replace, structured endoscopic and radiological follow-up, consistent with previous observations [5].

This study has limitations. Its single-centre observational design and modest sample size limit statistical power and may reduce the generalizability of the findings. Although overall follow-up was long and patients were routinely scheduled for postoperative assessments every 3–6 months, the actual timing and number of follow-up visits varied because of real-world factors, including individual scheduling needs and disruptions related to the COVID-19 pandemic. Consequently, residual bias related to irregular visit patterns cannot be excluded despite the use of mixed-effects modelling. The study population was heterogeneous with respect to tumour location and surgical management. Although this reflects routine clinical practice in sinonasal inverted papilloma, such heterogeneity may have influenced postoperative symptoms and recurrence patterns and may limit the generalizability of the findings. In addition, the single-centre setting may reflect local referral patterns, surgical expertise and follow-up practices, which may further limit the applicability of the findings to other institutions and patient populations. Due to the limited cohort size, meaningful subgroup analyses according to tumour location or surgical approach were not feasible. The most important limitation concerns the absence of prospectively collected preoperative PROMs. Retrospectively recalled preoperative SNOT-22 and WHOQOL-BREF scores were available only in a subset of patients and may be affected by recall bias, response-shift bias, and patients’ current postoperative health status. Therefore, paired pre–post comparisons were considered exploratory and hypothesis-generating, and they should not be interpreted as equivalent to comparisons based on prospectively collected baseline PROMs. In addition, SNOT-22 data were recorded and retained as total scores; item-level responses were not available for retrospective subdomain analysis. Although the total SNOT-22 score is a validated and commonly reported disease-specific outcome, the absence of item-level data may have obscured differential changes in specific symptom domains. Future studies should retain item-level SNOT-22 responses and examine longitudinal changes across individual symptom domains. Therefore, the primary inference of this study is based on postoperative longitudinal SNOT-22 trajectories. Selection bias is also possible, as analyses were restricted to patients with at least two follow-up visits and complete PROM data. Patients who were lost to follow-up or had insufficient postoperative assessments may have differed systematically from those included in the analyses, potentially limiting the representativeness of the study cohort. Baseline characteristics of excluded patients were not available for formal comparison with the analysed cohort.

## 5. Conclusions

Overall, our findings suggest that endoscopic management of IP is associated with favourable and stable long-term patient-reported outcomes. No single baseline demographic or tumour-related factor showed a clear association with postoperative SNOT-22 scores after accounting for follow-up time. These findings support postoperative counselling grounded in generally good long-term QoL while reinforcing the need for structured surveillance in this recurrent condition. Together with previous studies [19,20,21,22], our findings suggest that favourable postoperative QoL may be maintained over extended follow-up after endoscopic treatment of IP. Future prospective studies with systematically collected baseline PROMs are needed to confirm the exploratory pre–post findings.

## Figures and Tables

**Table 1:** Baseline characteristics of the study cohort. Baseline demographic and clinical characteristics of 69 patients with sinonasal inverted papilloma included in the study. Continuous variables are presented as mean (standard deviation), and categorical variables as number (percentage), unless otherwise stated. SNOT-22 refers to the postoperative disease-specific quality-of-life score.

Variable	N = 69 ^1^
Age, years	61.7 (12.5)
Sex	
Female	29/69 (42%)
Male	40/69 (58%)
Smoking status	31/69 (45%)
Krouse stage	
T1	5/69 (7%)
T2	40/69 (58%)
T3	22/69 (32%)
T4	2/69 (3%)
Previous sinonasal surgery ^2^	33/69 (48%)
Number of previous surgeries	
1	21/69 (30%)
2	6/69 (9%)
3	4/69 (6%)
≥7 ^3^	2/69 (3%)
Type of previous surgical approach	
Endoscopic	22/69 (32%)
Classical polypectomy	11/69 (16%)
Open approach	3/69 (4.3%)
Tumour debulking	3/69 (4.3%)
Hybrid approach	0/69 (0%)
Maxillectomy	0/69 (0%)
Follow-up duration, months	63.1 (27.8)
Recurrence	21/69 (30%)
Postoperative SNOT-22 score	25.4 (19.8)
Side	
Left	28/69 (41%)
Right	41/69 (59%)
Tumour location	
Maxillary sinus	41/69 (59%)
Ethmoid sinus	11/69 (17%)
Nasal cavity	9/69 (13%)
Frontal sinus	5/69 (7%)
Nasal septum	2/69 (3%)
Sphenoid sinus	1/69 (1%)

^1^ Values are presented as **mean (SD)** or **n/N (%)**. ^2^ Patients could have undergone more than one type of previous surgical procedure; therefore, categories of previous surgical approach are not mutually exclusive. ^3^ No patients had 4–6 previous surgeries; therefore, only the observed categories are presented.

**Table 2:** Univariable linear mixed-effects models for longitudinal SNOT-22 scores after endoscopic surgery. Results of univariable linear mixed-effects models assessing associations between selected clinical variables and longitudinal trajectories of SNOT-22 scores. Models included a patient-level random intercept to account for within-patient correlation. Time was included as a continuous covariate in each model. The β coefficients presented for clinical variables represent their estimated associations with postoperative SNOT-22 scores after accounting for follow-up time. Estimates were obtained using restricted maximum likelihood (REML); *p*-values were calculated using Satterthwaite’s approximation.

Variable	Beta ^1^	95% CI ^2^	*p*-Value
Sex			0.41
Female (ref. male)	3.6	−5.0 to 12.0	
Smoking (ref. non-smoker)	6.5	−2.0 to 15.0	0.18
Krouse stage (ordinal trend T1–T4)	−5.3	−12.0 to 1.2	0.10
Previous sinonasal surgery (ref. none)	−0.38	−8.9 to 8.1	0.93
Number of previous surgeries	1.9	−1.2 to 4.9	0.23
Previous surgical approach			
Endoscopic	−4.1	−13.0 to 4.9	0.36
Classical polypectomy	6.0	−5.5 to 17.0	0.30
Side			0.65
Right (ref. left)	2.0	−6.7 to 11.0	
Tumour location			
Maxillary sinus	−2.0	−11.0 to 6.6	0.64
Ethmoid sinus	−0.81	−12.0 to 11.0	0.89
Nasal cavity	7.4	−4.9 to 20.0	0.23

^1^ β coefficient represents the estimated effect of each clinical variable on postoperative SNOT-22 scores after accounting for follow-up time. For categorical variables, β represents the estimated difference relative to the reference category; for continuous or ordinal variables, β represents the estimated change associated with a one-unit increase. Time was modelled continuously in 6-month units. ^2^ CI = confidence interval.

**Table 3:** Exploratory comparison of retrospectively recalled preoperative and postoperative quality-of-life scores. Paired retrospectively recalled preoperative and postoperative values of disease-specific (SNOT-22) and generic (WHOQOL-BREF) quality-of-life measures in the exploratory subgroup. Preoperative values were obtained during follow-up using a retrospective pre-test approach and should not be interpreted as prospectively collected baseline data.

Variable	Preoperative, N = 34 ^1^	Postoperative, N = 34 ^1^	*p* ^2^
SNOT-22	39.5 (16.5, 50.8)	24.0 (12.8, 39.8)	**0.037**
WHOQOL-BREF Q1: Overall quality of life	3.0 (3.0, 4.0)	4.0 (3.0, 4.0)	0.10
WHOQOL-BREF Q2: General condition	3.0 (2.0, 4.0)	3.0 (3.0, 4.0)	0.18
WHOQOL-BREF: Physical domain	22.0 (20.3, 24.0)	23.0 (21.0, 26.0)	**0.024**
WHOQOL-BREF: Psychological domain	20.0 (19.0, 23.0)	21.5 (19.0, 23.0)	**0.041**
WHOQOL-BREF: Social relationships domain	11.0 (10.0, 12.0)	11.0 (10.0, 12.0)	0.99
WHOQOL-BREF: Environmental domain	29.5 (27.0, 32.0)	31.0 (28.0, 33.8)	**0.03**

^1^ Values are presented as median (interquartile range). ^2^ Wilcoxon signed-rank test. Statistically significant *p*-values (<0.05) are shown in bold.

## Data Availability

The original contributions presented in this study are included in the article/Supplementary Materials. Further inquiries can be directed to the corresponding author.

## Supplementary Materials

The following supporting information can be downloaded at: https://www.mdpi.com/article/10.3390/medicina62081567/s1, Figure S1. Longitudinal postoperative SNOT-22 trajectories stratified by sex; Figure S2. Longitudinal postoperative SNOT-22 trajectories stratified by smoking status; Figure S3. Longitudinal postoperative SNOT-22 trajectories stratified by history of previous sinonasal surgery; Figure S4. Longitudinal postoperative SNOT-22 trajectories stratified by recurrence status; Figure S5. Longitudinal postoperative SNOT-22 trajectories stratified by side of involvement; Table S1. Baseline characteristics stratified by sex; Table S2. Baseline characteristics stratified by smoking status; Table S3. Baseline characteristics stratified by side of involvement; Table S4. Baseline characteristics stratified by history of previous sinonasal surgery; Table S5. Baseline characteristics stratified by recurrence status; Table S6. Change in quality of life (SNOT-22, WHOQOL-BREF) after inverted papilloma surgery; Table S7. Change in quality of life (SNOT-22, WHOQOL-BREF) after surgery stratified by sex; Table S8. Change in quality of life (SNOT-22, WHOQOL-BREF) after surgery stratified by smoking status; Table S9. Change in quality of life (SNOT-22, WHOQOL-BREF) after surgery stratified by history of previous sinonasal surgery; Table S10. Change in quality of life (SNOT-22, WHOQOL-BREF) after surgery stratified by recurrence. File S1: STROBE Checklist.

## Abbreviations

The following abbreviations are used in this manuscript:

CI	Confidence interval
CRSwNP	Chronic rhinosinusitis with nasal polyps
CT	Computed tomography
ENT	Otorhinolaryngology
GAM	Generalised additive model
HRQoL	Health-related quality of life
IP	Inverted papilloma
IQR	Interquartile range
LMM	Linear mixed-effects model
MRI	Magnetic resonance imaging
PROM	Patient-reported outcome measure
QoL	Quality of life
REML	Restricted maximum likelihood
SCC	Squamous cell carcinoma
SD	Standard deviation
SNOT-22	Sino-Nasal Outcome Test-22
WHO	World Health Organization
WHOQOL-BREF	World Health Organization Quality of Life-BREF
