# Supplementary material for: Long-Term Postoperative Patient-Reported Quality of Life After Endoscopic Surgery for Sinonasal Inverted Papilloma: A Longitudinal Cohort Study
Source: Medicina (Kaunas). 2026 Aug 15;62(8):1567. doi: 10.3390/medicina62081567 (PMC13515541; doi:10.3390/medicina62081567)
Supplement: Supplementary file 1 [file medicina-62-01567-s001.zip › Supplementary tables.pdf]

Supplementary tables:

Table S1. Baseline characteristics stratified by sex

**Variable**	**Female, N = 29[1]**	**Male, N = 39[1]**	**p[2]**
Age [years]	59.00 (15.00)	63.46 (10.09)	0.20
Smoking	12 / 29 (41.4%)	18 / 39 (46.2%)	0.80
**Krouse stage**			0.30
T1	2 / 29 (6.9%)	3 / 39 (7.7%)	
T2	15 / 29 (51.7%)	25 / 39 (64.1%)	
T3	12 / 29 (41.4%)	9 / 39 (23.1%)	
T4	0 / 29 (0.0%)	2 / 39 (5.1%)	
Previous sinonasal surgery	16 / 29 (55.2%)	16 / 39 (41.0%)	0.30
**Number of previous surgeries**			0.40
1	8 / 29 (27.6%)	12 / 39 (30.8%)	
2	4 / 29 (13.8%)	2 / 39 (5.1%)	
3	3 / 29 (10.3%)	1 / 39 (2.6%)	
7	1 / 29 (3.4%)	1 / 39 (2.6%)	
Endoscopic approach	11 / 29 (37.9%)	10 / 39 (25.6%)	0.30
Classical polypectomy	4 / 29 (13.8%)	7 / 39 (17.9%)	0.70
Open approach	2 / 29 (6.9%)	1 / 39 (2.6%)	0.60
Lesion removal only	2 / 29 (6.9%)	1 / 39 (2.6%)	0.60
Follow-up duration [months]	54.86 (28.47)	69.23 (26.33)	**0.038**
Recurrence	11 / 29 (37.9%)	9 / 39 (23.1%)	0.30
Postoperative SNOT-22	26.45 (22.62)	25.08 (17.81)	0.80
**Side**			0.20
Left	9 / 29 (31.0%)	19 / 39 (48.7%)	

Variable	Female, N = 29[1]	Male, N = 39[1]	p[2]
Right	20 / 29 (69.0%)	20 / 39 (51.3%)	
Maxillary sinus	19 / 29 (65.5%)	22 / 39 (56.4%)	0.50
Ethmoid sinus	4 / 29 (13.8%)	6 / 39 (15.4%)	>0.90
Nasal cavity	3 / 29 (10.3%)	6 / 39 (15.4%)	0.70
Frontal sinus	3 / 29 (10.3%)	2 / 39 (5.1%)	0.60
Nasal septum	0 / 29 (0.0%)	2 / 39 (5.1%)	0.50
Sphenoid sinus	0 / 29 (0.0%)	1 / 39 (2.6%)	>0.90

[1] Mean (SD) or n / N (%)
[2] Welch's t-test or Fisher's exact test

Table S2. Baseline characteristics stratified by smoking status

Variable	Non-smoker, N = 38[1]	Smoker, N = 31[1]	p[2]
Age [years]	61.63 (14.45)	61.71 (9.71)	>0.90
**Sex**			0.80
Female	17 / 38 (44.7%)	12 / 30 (40.0%)	
Male	21 / 38 (55.3%)	18 / 30 (60.0%)	
**Krouse stage**			0.20
T1	3 / 38 (7.9%)	2 / 31 (6.5%)	
T2	20 / 38 (52.6%)	20 / 31 (64.5%)	
T3	15 / 38 (39.5%)	7 / 31 (22.6%)	
T4	0 / 38 (0.0%)	2 / 31 (6.5%)	
Previous sinonasal surgery	17 / 38 (44.7%)	16 / 31 (51.6%)	0.80
**Number of previous surgeries**			0.30
1	12 / 38 (31.6%)	9 / 31 (29.0%)	

Variable	Non-smoker, N = 38[1]	Smoker, N = 31[1]	p[2]
2	1 / 38 (2.6%)	5 / 31 (16.1%)	
3	3 / 38 (7.9%)	1 / 31 (3.2%)	
7	1 / 38 (2.6%)	1 / 31 (3.2%)	
Endoscopic approach	12 / 38 (31.6%)	10 / 31 (32.3%)	>0.90
Classical polypectomy	4 / 38 (10.5%)	7 / 31 (22.6%)	0.20
Lesion removal only	2 / 38 (5.3%)	1 / 31 (3.2%)	>0.90
Open approach	1 / 38 (2.6%)	2 / 31 (6.5%)	0.60
Follow-up duration [months]	61.53 (27.95)	65.00 (27.90)	0.60
Recurrence	11 / 38 (28.9%)	10 / 31 (32.3%)	>0.90
Postoperative SNOT-22	23.50 (18.67)	27.81 (21.17)	0.30
**Side**			0.09
Left	12 / 38 (31.6%)	16 / 31 (51.6%)	
Right	26 / 38 (68.4%)	15 / 31 (48.4%)	
Maxillary sinus	23 / 38 (60.5%)	18 / 31 (58.1%)	>0.90
Ethmoid sinus	6 / 38 (15.8%)	5 / 31 (16.1%)	>0.90
Nasal cavity	3 / 38 (7.9%)	6 / 31 (19.4%)	0.20
Frontal sinus	3 / 38 (7.9%)	2 / 31 (6.5%)	>0.90
Nasal septum	2 / 38 (5.3%)	0 / 31 (0.0%)	0.50
Sphenoid sinus	1 / 38 (2.6%)	0 / 31 (0.0%)	>0.90

[1] Mean (SD) or n / N (%)

[2] Welch’s t-test or Fisher’s exact test

Table S3. Baseline characteristics stratified by side of involvement

Variable	Left, N = 28[1]	Right, N = 41[1]	p[2]
Age [years]	60.64 (12.26)	62.37 (12.69)	0.60
**Sex**			0.20
Female	9 / 28 (32.1%)	20 / 40 (50.0%)	
Male	19 / 28 (67.9%)	20 / 40 (50.0%)	
Smoking	16 / 28 (57.1%)	15 / 41 (36.6%)	0.14
**Krouse stage**			0.30
T1	3 / 28 (10.7%)	2 / 41 (4.9%)	
T2	16 / 28 (57.1%)	24 / 41 (58.5%)	
T3	7 / 28 (25.0%)	15 / 41 (36.6%)	
T4	2 / 28 (7.1%)	0 / 41 (0.0%)	
Previous sinonasal surgery	11 / 28 (39.3%)	22 / 41 (53.7%)	0.30
**Number of previous surgeries**			0.20
1	5 / 28 (17.9%)	16 / 41 (39.0%)	
2	3 / 28 (10.7%)	3 / 41 (7.3%)	
3	1 / 28 (3.6%)	3 / 41 (7.3%)	
7	2 / 28 (7.1%)	0 / 41 (0.0%)	
Endoscopic approach	8 / 28 (28.6%)	14 / 41 (34.1%)	0.80
Classical polypectomy	4 / 28 (14.3%)	7 / 41 (17.1%)	>0.90
Open approach	0 / 28 (0.0%)	3 / 41 (7.3%)	0.30
Lesion removal only	2 / 28 (7.1%)	1 / 41 (2.4%)	0.60
Follow-up duration [months]	64.68 (28.37)	62.00 (27.67)	0.70
Recurrence	7 / 28 (25.0%)	14 / 41 (34.1%)	0.60
Postoperative SNOT-22	24.61 (20.84)	26.00 (19.29)	0.70
Maxillary sinus	15 / 28 (53.6%)	26 / 41 (63.4%)	0.50

Variable	Left, N = 28[1]	Right, N = 41[1]	p[2]
Ethmoid sinus	3 / 28 (10.7%)	8 / 41 (19.5%)	0.50
Nasal cavity	5 / 28 (17.9%)	4 / 41 (9.8%)	0.50
Frontal sinus	3 / 28 (10.7%)	2 / 41 (4.9%)	0.40
Nasal septum	1 / 28 (3.6%)	1 / 41 (2.4%)	>0.90
Sphenoid sinus	1 / 28 (3.6%)	0 / 41 (0.0%)	0.40

[1] Mean (SD) or n / N (%)

[2] Welch's t-test or Fisher's exact test

Table S4. Baseline characteristics stratified by history of previous sinonasal surgery

Variable	No, N = 36[1]	Yes, N = 33[1]	p[2]
Age [years]	61.06 (13.34)	62.33 (11.59)	0.70
**Sex**			0.30
Female	13 / 36 (36.1%)	16 / 32 (50.0%)	
Male	23 / 36 (63.9%)	16 / 32 (50.0%)	
Smoking	15 / 36 (41.7%)	16 / 33 (48.5%)	0.80
**Krouse stage**			0.50
T1	3 / 36 (8.3%)	2 / 33 (6.1%)	
T2	20 / 36 (55.6%)	20 / 33 (60.6%)	
T3	13 / 36 (36.1%)	9 / 33 (27.3%)	
T4	0 / 36 (0.0%)	2 / 33 (6.1%)	
Follow-up duration [months]	56.03 (28.12)	70.79 (25.64)	**0.025**
Recurrence	8 / 36 (22.2%)	13 / 33 (39.4%)	0.20
Postoperative SNOT-22	25.22 (20.97)	25.67 (18.75)	0.80
**Side**			0.30

Variable	No, N = 36[1]	Yes, N = 33[1]	p[2]
Left	17 / 36 (47.2%)	11 / 33 (33.3%)	
Right	19 / 36 (52.8%)	22 / 33 (66.7%)	
Maxillary sinus	20 / 36 (55.6%)	21 / 33 (63.6%)	0.50
Ethmoid sinus	2 / 36 (5.6%)	9 / 33 (27.3%)	**0.038**
Nasal cavity	8 / 36 (22.2%)	1 / 33 (3.0%)	**0.03**
Frontal sinus	4 / 36 (11.1%)	1 / 33 (3.0%)	0.40
Nasal septum	1 / 36 (2.8%)	1 / 33 (3.0%)	>0.90
Sphenoid sinus	1 / 36 (2.8%)	0 / 33 (0.0%)	>0.90

[1] Mean (SD) or n / N (%)
[2] Welch's t-test or Fisher's exact test

Table S5. Baseline characteristics stratified by recurrence status

Variable	No recurrence, N = 48[1]	Recurrence, N = 21[1]	p[2]
Age [years]	62.08 (12.90)	60.71 (11.63)	0.60
**Sex**			0.30
Female	18 / 48 (37.5%)	11 / 20 (55.0%)	
Male	30 / 48 (62.5%)	9 / 20 (45.0%)	
Smoking	21 / 48 (43.8%)	10 / 21 (47.6%)	>0.90
**Krouse stage**			0.30
T1	5 / 48 (10.4%)	0 / 21 (0.0%)	
T2	29 / 48 (60.4%)	11 / 21 (52.4%)	
T3	13 / 48 (27.1%)	9 / 21 (42.9%)	
T4	1 / 48 (2.1%)	1 / 21 (4.8%)	
Previous sinonasal surgery	20 / 48 (41.7%)	13 / 21 (61.9%)	0.20

Variable	No recurrence, N = 48[1]	Recurrence, N = 21[1]	p[2]
**Number of previous surgeries**			**0.002**
1	17 / 48 (35.4%)	4 / 21 (19.0%)	
2	1 / 48 (2.1%)	5 / 21 (23.8%)	
3	2 / 48 (4.2%)	2 / 21 (9.5%)	
7	0 / 48 (0.0%)	2 / 21 (9.5%)	
Endoscopic approach	11 / 48 (22.9%)	11 / 21 (52.4%)	**0.024**
Classical polypectomy	8 / 48 (16.7%)	3 / 21 (14.3%)	>0.90
Open approach	2 / 48 (4.2%)	1 / 21 (4.8%)	>0.90
Lesion removal only	2 / 48 (4.2%)	1 / 21 (4.8%)	>0.90
Follow-up duration [months]	64.63 (28.19)	59.57 (27.16)	0.50
Postoperative SNOT-22	24.65 (19.93)	27.24 (19.85)	0.50
**Side**			0.60
Left	21 / 48 (43.8%)	7 / 21 (33.3%)	
Right	27 / 48 (56.3%)	14 / 21 (66.7%)	
Maxillary sinus	28 / 48 (58.3%)	13 / 21 (61.9%)	0.80
Ethmoid sinus	6 / 48 (12.5%)	5 / 21 (23.8%)	0.50
Nasal cavity	8 / 48 (16.7%)	1 / 21 (4.8%)	0.30
Frontal sinus	3 / 48 (6.3%)	2 / 21 (9.5%)	0.60
Nasal septum	2 / 48 (4.2%)	0 / 21 (0.0%)	>0.90
Sphenoid sinus	1 / 48 (2.1%)	0 / 21 (0.0%)	>0.90

[1] Mean (SD) or n / N (%)

[2] Welch's t-test or Fisher's exact test

Table S6. Change in quality of life (SNOT-22, WHOQOL-BREF) after inverted papilloma surgery

Variable	Preoperative, N = 34[1]	Postoperative, N = 34[1]	p[2]
SNOT-22	39.5 (16.5, 50.8)	24.0 (12.8, 39.8)	0.037
WHOQOL-BREF Q1: Overall quality of life	3.0 (3.0, 4.0)	4.0 (3.0, 4.0)	0.10
WHOQOL-BREF Q2: General health	3.0 (2.0, 4.0)	3.0 (3.0, 4.0)	0.18
WHOQOL-BREF: Physical domain	22.0 (20.3, 24.0)	23.0 (21.0, 26.0)	0.024
WHOQOL-BREF: Psychological domain	20.0 (19.0, 23.0)	21.5 (19.0, 23.0)	0.041
WHOQOL-BREF: Social relationships domain	11.0 (10.0, 12.0)	11.0 (10.0, 12.0)	0.99
WHOQOL-BREF: Environmental domain	29.5 (27.0, 32.0)	31.0 (28.0, 33.8)	0.03

[1] Median (IQR)
[2] Wilcoxon signed-rank test
p-values < 0.05 were considered statistically significant.

Table S7. Change in quality of life (SNOT-22, WHOQOL-BREF) after surgery stratified by sex

Variable	Female: Preoperative, N = 8[1]	Female: Postoperative, N = 8[1]	p[2]	Male: Preoperative, N = 25[1]	Male: Postoperative, N = 25[1]	p[2]
SNOT-22	43.5 (40.8, 51.8)	40.5 (5.0, 58.5)	0.19	29.0 (16.0, 51.0)	23.0 (15.0, 34.0)	0.166
WHOQOL-BREF Q1: Overall quality of life	3.0 (3.0, 4.0)	3.5 (3.0, 4.0)	0.80	3.0 (3.0, 4.0)	4.0 (3.0, 4.0)	0.10
WHOQOL-BREF Q2:	3.0 (3.0, 4.0)	3.0 (3.0, 4.0)	>0.99	3.0 (2.0, 4.0)	3.0 (3.0, 4.0)	0.023

Variable	Female: Preoperative, N = 8[1]	Female: Postoperative, N = 8[1]	p[2]	Male: Preoperative, N = 25[1]	Male: Postoperative, N = 25[1]	p[2]
General health						
WHOQOL-BREF: Physical domain	22.0 (20.8, 22.5)	23.0 (22.3, 26.3)	0.08	22.0 (20.0, 24.0)	22.0 (21.0, 26.0)	0.05
WHOQOL-BREF: Psychological domain	21.0 (20.0, 22.3)	22.5 (19.8, 23.3)	0.13	20.0 (19.0, 23.0)	21.0 (19.0, 23.0)	0.13
WHOQOL-BREF: Social relationships domain	12.0 (11.0, 12.0)	12.0 (10.5, 13.0)	>0.99	11.0 (10.0, 12.0)	11.0 (10.0, 12.0)	0.72
WHOQOL-BREF: Environmental domain	30.0 (27.8, 32.0)	32.0 (28.8, 35.5)	0.014	29.0 (27.0, 32.0)	31.0 (28.0, 32.0)	0.18

[1] Median (IQR)
[2] Wilcoxon signed-rank test
p-values < 0.05 were considered statistically significant.

Table S8. Change in quality of life (SNOT-22, WHOQOL-BREF) after surgery stratified by smoking status

Variable	Non-smoker: Preoperative, N = 19[1]	Non-smoker: Postoperative, N = 19[1]	p[2]	Smoker: Preoperative, N = 15[1]	Smoker: Postoperative, N = 15[1]	p[2]
SNOT-22	34.0 (15.5, 47.0)	18.0 (9.0, 36.5)	0.18	43.0 (28.5, 64.5)	28.0 (23.0, 46.0)	0.08
WHOQOL-BREF Q1:	4.0 (3.0, 4.0)	4.0 (3.5, 4.0)	0.30	3.0 (3.0, 3.5)	3.0 (3.0, 4.0)	0.24

Variable	Non-smoker: Preoperative, N = 19[1]	Non-smoker: Postoperative, N = 19[1]	p[2]	Smoker: Preoperative, N = 15[1]	Smoker: Postoperative, N = 15[1]	p[2]
Overall quality of life						
WHOQOL-BREF Q2: General health	3.0 (2.5, 4.0)	3.0 (3.0, 4.0)	0.30	3.0 (2.0, 3.0)	3.0 (3.0, 3.0)	0.30
WHOQOL-BREF: Physical domain	22.0 (21.0, 23.5)	23.0 (21.0, 25.5)	0.26	22.0 (20.0, 24.5)	23.0 (21.0, 26.5)	0.046
WHOQOL-BREF: Psychological domain	20.0 (18.5, 23.0)	22.0 (19.5, 23.0)	0.23	20.0 (19.0, 22.5)	21.0 (19.0, 24.0)	0.08
WHOQOL-BREF: Social relationships domain	11.0 (10.5, 12.0)	11.0 (10.0, 12.5)	0.42	11.0 (9.5, 12.5)	11.0 (9.0, 12.0)	0.56
WHOQOL-BREF: Environmental domain	30.0 (27.0, 32.0)	31.0 (28.0, 33.5)	0.25	28.0 (26.5, 31.0)	31.0 (28.5, 33.0)	0.06

[1] Median (IQR)
[2] Wilcoxon signed-rank test
p-values < 0.05 were considered statistically significant.

Table S9. Change in quality of life (SNOT-22, WHOQOL-BREF) after surgery stratified by history of previous sinonasal surgery

Variable	No previous surgery: Preoperative, N = 14[1]	No previous surgery: Postoperative, N = 14[1]	p[2]	Previous surgery: Preoperative, N = 20[1]	Previous surgery: Postoperative, N = 20[1]	p[2]
SNOT-22	34.5 (16.0, 47.0)	27.5 (17.5, 43.8)	0.53	39.5 (25.5, 57.0)	22.0 (12.0, 35.3)	0.046
WHOQOL-BREF Q1: Overall quality of life	3.0 (3.0, 3.8)	3.5 (3.0, 4.0)	0.12	4.0 (3.0, 4.0)	4.0 (3.0, 4.0)	0.59
WHOQOL-BREF Q2: General health	3.0 (2.3, 4.0)	3.5 (3.0, 4.0)	0.13	3.0 (2.0, 4.0)	3.0 (3.0, 3.3)	0.53
WHOQOL-BREF: Physical domain	21.0 (18.8, 22.8)	22.0 (19.3, 25.0)	0.17	22.5 (20.8, 24.3)	23.0 (22.8, 26.0)	0.09
WHOQOL-BREF: Psychological domain	21.0 (19.0, 22.8)	20.5 (19.0, 22.8)	0.93	20.0 (19.0, 23.0)	22.0 (20.0, 23.3)	0.016
WHOQOL-BREF: Social relationships domain	11.0 (10.3, 12.0)	11.0 (9.0, 11.8)	0.38	11.0 (10.0, 12.0)	11.5 (10.8, 12.0)	0.34
WHOQOL-BREF: Environmental domain	29.5 (27.0, 32.0)	31.0 (28.0, 33.5)	0.67	29.5 (27.0, 32.0)	31.0 (29.0, 33.3)	0.015

[1] Median (IQR)

[2] Wilcoxon signed-rank test

p-values < 0.05 were considered statistically significant.

Table S10. Change in quality of life (SNOT-22, WHOQOL-BREF) after surgery stratified by recurrence

Variable	No recurrence: Preoperative, N = 26[1]	No recurrence: Postoperative, N = 26[1]	p[2]	Recurrence: Preoperative, N = 8[1]	Recurrence: Postoperative, N = 8[1]	p[2]
SNOT-22	43.5 (18.5, 50.8)	26.5 (15.0, 43.8)	0.13	29.0 (15.8, 42.8)	19.5 (11.5, 26.3)	0.08
WHOQOL-BREF Q1: Overall quality of life	3.0 (3.0, 4.0)	4.0 (3.0, 4.0)	0.09	4.0 (3.0, 4.0)	4.0 (3.0, 4.0)	>0.99
WHOQOL-BREF Q2: General health	3.0 (2.3, 4.0)	3.0 (3.0, 4.0)	0.07	3.5 (2.0, 4.0)	3.0 (3.0, 3.3)	>0.99
WHOQOL-BREF: Physical domain	22.0 (20.3, 24.0)	23.0 (21.3, 26.0)	0.007	22.5 (20.8, 23.3)	22.0 (20.8, 23.0)	0.83
WHOQOL-BREF: Psychological domain	20.5 (18.3, 22.8)	21.5 (19.3, 23.0)	0.03	20.0 (19.0, 23.0)	21.0 (18.8, 22.3)	0.85
WHOQOL-BREF: Social relationships domain	11.0 (10.0, 12.0)	11.0 (9.3, 12.0)	0.87	12.0 (10.8, 12.5)	11.5 (11.0, 12.3)	>0.99
WHOQOL-BREF: Environmental domain	29.5 (27.0, 32.0)	31.0 (29.0, 32.8)	0.03	29.0 (27.0, 32.5)	30.0 (27.5, 34.0)	0.75

[1] Median (IQR)

[2] Wilcoxon signed-rank test

p-values < 0.05 were considered statistically significant.
