# Supplementary material for: Long-Term Postoperative Patient-Reported Quality of Life After Endoscopic Surgery for Sinonasal Inverted Papilloma: A Longitudinal Cohort Study
Source: Medicina (Kaunas). 2026 Aug 15;62(8):1567. doi: 10.3390/medicina62081567 (PMC13515541; doi:10.3390/medicina62081567)
Supplement: Supplementary file 1 [file medicina-62-01567-s001.zip › Supplementary_File_S1_STROBE_Checklist_corrected.pdf]

STROBE Statement—Checklist of items that should be included in reports of cohort studies

Manuscript: Long-Term Postoperative Patient-Reported Quality of Life After Endoscopic Surgery for Sinonasal Inverted Papilloma: A Longitudinal Cohort Study

Section/Topic	Item No.	Recommendation	Page No./Section
Title and abstract	1(a)	Indicate the study's design with a commonly used term in the title or the abstract	Title and Abstract, p. 1
	1(b)	Provide in the abstract an informative and balanced summary of what was done and what was found	Abstract, p. 1
Introduction – Background/rationale	2	Explain the scientific background and rationale for the investigation being reported	Introduction, p. 2
Introduction – Objectives	3	State specific objectives, including any prespecified hypotheses	Introduction, pp. 2–3
Methods – Study design	4	Present key elements of study design early in the paper	Section 2.1, p. 3
Methods – Setting	5	Describe the setting, locations, and relevant dates, including periods of recruitment, exposure, follow-up, and data collection	Sections 2.1–2.2, p. 3 (follow-up through 30 September 2025)
Methods – Participants	6(a)	Give the eligibility criteria, and the sources and methods of selection of participants. Describe methods of follow-up	Sections 2.1–2.2, p. 3; Figure 1, p. 4
	6(b)	For matched studies, give matching criteria and number of exposed and unexposed	N/A – not a matched study
Methods – Variables	7	Clearly define all outcomes, exposures, predictors, potential confounders, and effect modifiers. Give diagnostic criteria, if applicable	Sections 2.3–2.4, pp. 4–5
Methods – Data sources/measurement	8*	For each variable of interest, give sources of data and details of methods of assessment (measurement). Describe comparability of assessment methods if there is more than one group	Sections 2.1–2.4, pp. 3–5
Methods – Bias	9	Describe any efforts to address potential sources of bias	Sections 2.2 and 2.5, pp. 3 and 5; Discussion/Limitations, pp. 10–11
Methods – Study size	10	Explain how the study size was arrived at	Section 2.1, p. 3 (eligible-patient cohort; no a priori sample-size calculation)
Methods – Quantitative variables	11	Explain how quantitative variables were handled in the analyses. If applicable, describe which groupings were chosen and why	Sections 2.4–2.5, p. 5
Methods – Statistical methods	12(a)	Describe all statistical methods, including those used to control for confounding	Section 2.5, p. 5
	12(b)	Describe any methods used to examine subgroups and interactions	Section 2.5, p. 5
	12(c)	Explain how missing data were addressed	Section 2.5, p. 5
	12(d)	If applicable, explain how loss to follow-up was addressed	Section 2.1, p. 3; Figure 1, p. 4; Discussion/Limitations, pp. 10–11
	12(e)	Describe any sensitivity analyses	N/A – no sensitivity analyses were performed
Results – Participants	13(a)*	Report numbers of individuals at each stage of study—eg numbers potentially eligible, examined for eligibility, confirmed eligible, included in the study, completing follow-up, and analysed	Section 2.1, p. 3; Figure 1, p. 4; Section 3.1, pp. 6–7
	13(b)*	Give reasons for non-participation at each stage	Section 2.1, p. 3; Figure 1, p. 4
	13(c)*	Consider use of a flow diagram	Figure 1, p. 4
Results – Descriptive data	14(a)*	Give characteristics of study participants (eg demographic, clinical, social) and information on exposures and potential confounders	Section 3.1 and Table 1, pp. 6–7
	14(b)*	Indicate number of participants with missing data for each variable of interest	Sections 2.2 and 2.5, pp. 3 and 5; Table 1, pp. 6–7; Section 3.3, pp. 8–9 (paired subgroup n = 34)
	14(c)*	Summarise follow-up time (eg, average and total amount)	Section 3.1 and Table 1, pp. 6–7
Results – Outcome data	15*	Report numbers of outcome events or summary measures over time	Sections 3.1–3.3, pp. 6–9; Tables 1–3; Figure 2
Results – Main results	16(a)	Give unadjusted estimates and, if applicable, confounder-adjusted estimates and their precision (eg, 95% confidence interval). Make clear which confounders were adjusted for and why they were included	Section 3.2 and Table 2, pp. 7–8 (univariable estimates with 95% CIs; no multivariable-adjusted model)
	16(b)	Report category boundaries when continuous variables were categorized	N/A – continuous variables were not categorized for the main analyses
	16(c)	If relevant, consider translating estimates of relative risk into absolute risk for a meaningful time period	N/A – relative-risk estimates were not used
Results – Other analyses	17	Report other analyses done—eg analyses of subgroups and interactions, and sensitivity analyses	Section 3.3, pp. 8–9; Table 3; Supplementary Tables S6–S10; Supplementary Figures S1–S5
Discussion – Key results	18	Summarise key results with reference to study objectives	Discussion, pp. 9–10
Discussion – Limitations	19	Discuss limitations of the study, taking into account sources of potential bias or imprecision. Discuss both direction and magnitude of any potential bias	Discussion/Limitations, pp. 10–11

Section/Topic	Item No.	Recommendation	Page No./Section
Discussion – Interpretation	20	Give a cautious overall interpretation of results considering objectives, limitations, multiplicity of analyses, results from similar studies, and other relevant evidence	Discussion, pp. 9–11
Discussion – Generalisability	21	Discuss the generalisability (external validity) of the study results	Discussion/Limitations, pp. 10–11
Other information – Funding	22	Give the source of funding and the role of the funders for the present study and, if applicable, for the original study on which the present article is based	Funding statement, p. 11

* Give information separately for exposed and unexposed groups, where applicable.

Page references correspond to the current 13-page manuscript proof supplied for final proofreading.

Supplementary File S1. Completed STROBE checklist for cohort studies.
